# Labouring women who used a birthing pool in obsteric units in Italy: prospective observational study

**DOI:** 10.1186/1471-2393-14-17

**Published:** 2014-01-14

**Authors:** Jane Henderson, Ethel E Burns, Anna L Regalia, Giovanna Casarico, Mary G Boulton, Lesley A Smith

**Affiliations:** 1 Faculty of Health and Life Sciences, Oxford Brookes University, Jack Straws Lane, Oxford OX3 0FL, UK; 2 Department of Obstetrics and Gynaecology, University of Milano-Bicocca, Monza, Italy

**Keywords:** Observational comparative study, Birthing pool, Waterbirth, Maternal and neonatal outcomes

## Abstract

**Background:**

For women at low risk of childbirth complications, water immersion during labour is a care option in many high income countries. Our aims were (a) to describe maternal characteristics, intrapartum events, interventions, maternal and neonatal outcomes for all women who used a birthing pool during labour who either had a waterbirth or left the pool and had a landbirth, and for the subgroup of women who had a waterbirth in 19 obstetric units, and (b) to compare maternal characteristics, intrapartum events, interventions, and maternal and neonatal outcomes for women who used a birthing pool with a control group of women who did not use a birthing pool for whom we prospectively collected data in a single centre.

**Methods:**

Prospective observational study in 19 Italian obstetric units 2002-2005. Participants were: (a) 2,505 women in labour using a birthing pool in 19 obstetric units; and (b) 114 women in labour using a birthing pool and 459 women who did not use a birthing pool in one obstetric unit. Descriptive statistics were calculated for the sample as a whole and, separately, for those women who gave birth in water. Categorical data were compared using Chi square statistics and continuous data by T-tests.

**Results:**

Overall, 95.6% of women using a birthing pool had a spontaneous vertex delivery, 63.9% of which occurred in water. Half of nulliparas and three quarters of multiparas delivered in water. Adverse maternal and neonatal outcomes were rare. There were two cases of umbilical cord snap with waterbirth. Compared with controls, significantly more women who used a birthing pool adopted an upright birth position, had hands off delivery technique, and a physiological third stage. Significantly fewer nulliparas had an episiotomy, and more had a second degree perineal tear, with no evidence of a difference for extensive perineal tears.

**Conclusions:**

Birthing pool use was associated with spontaneous vaginal birth. The increase in second degree tears was balanced by fewer episiotomies. Undue umbilical cord traction should be avoided during waterbirth.

## Background

The use of water immersion to ease the discomfort and pain of labour is well established. It was popularised by Michel Odent in the 1980s [1] and has gradually increased in popularity and acceptance in most high income countries [1-4]. In Italy, birthing pools were introduced in 1990 but are considered a marginal care option. Italian intrapartum care is highly medicalised with the fourth highest global Caesarean section rate (38%) [5,6]. Midwives exercise less autonomy than in the UK [7]; it is common practice for an obstetrician to review a woman presenting in labour before allocating her care to a midwife, and for an obstetrician and paediatrician to be present at the birth.

Benefits to women of labouring in water include buoyancy and ease of movement facilitating women to maximise pelvic diameters which may lead to improved fetal flexion and easier delivery [8]. The warm water increases maternal relaxation reducing pain perception [9], and may lead to improved uterine perfusion and reduced blood pressure [10]. Women who use a birthing pool report an increased sense of control and satisfaction with the experience of birth [9,11,12].

Whilst labouring in water is generally considered safe for women without complications [13,14], concerns have been expressed about the safety of giving birth in water [3]. In particular snapped umbilical cord [15,16], and extensive perineal trauma [17]. Individual case reports involving adverse neonatal events [18-20] have contributed to some European countries banning waterbirth.

Another safety concern is the relationship between waterbirth, management of the third stage of labour and blood loss at delivery. Active management of the third stage of labour is recommended for all women, irrespective of their risk profile, however waterbirth usually involves physiological management. Active management comprises umbilical cord clamping and cutting, an oxytocic injection, and placental delivery by controlled cord traction. Physiological management involves delayed or no cord clamping, no oxytocic injection and placenta delivery by maternal effort. A Cochrane review showed fewer women had a primary postpartum haemorrhage (PPH) with active management. However, the evidence is inconclusive for women at low risk of childbirth complications as the randomised controlled trials (RCTs) involved women of a mixed risk profile [21]. A large observational study has suggested that physiological management does not increase the risk of PPH for women with straightforward pregnancy [22].

A recent Cochrane review of 12 trials (3,243 women) evaluating labour and delivery in water has reported reassuringly low rates of maternal and neonatal infection, low Apgar scores and neonatal unit admissions; adverse outcomes were not increased in women using birthing pools [9]. The authors also reported significant reductions in the need for epidural, spinal and paracervical analgesia, reduced duration of the first stage of labour, and increased maternal satisfaction in women labouring in water compared with women labouring out of water. There were no differences in mode of delivery, rates of augmentation or perineal trauma. However, only three of the trials included in the review investigated the effects of immersion during the second stage of labour. A recent UK prospective study reported on interventions and outcomes for 8,924 women who laboured in water, 5192 of whom gave birth in water [14]. Eighty nine percent of women who used a birthing pool had a spontaneous birth, and there were few maternal and neonatal adverse events.

The primary objective of this study was (a) to describe the maternal characteristics, intrapartum events, interventions, and maternal and neonatal outcomes for all women who used a birthing pool during labour who either had a waterbirth or left the pool and had a landbirth, and for the subgroup of women who had a waterbirth in 19 obstetric units. A secondary objective (b) was to compare maternal characteristics, intrapartum events, interventions, and maternal and neonatal outcomes for women who used a birthing pool with a control group of women who were eligible to use the birthing pool but did not and for whom we collected prospective data in one obstetric unit.

## Methods

A prospective observational study design was used. When the study started in 2001 there were 559 maternity units in Italy, 50 of which had a birthing pool. Four of these were in private hospitals but the remaining 46 public obstetric units were invited to participate in this study. These units are representative of the range of obstetric units in Italy. They varied in size from less than 500 to over 2,500 births per year. Most were located in the north of the country because few of the southern units had birthing pools (Additional file 1: Table S1). Forty-six units were contacted by phone in early 2002, of which 35 expressed an interest in participating and were sent information about the study. Of these, 21 units agreed to participate. These data were collected as part of an international study, for which the findings for the UK have been published [14].

A three to six month pilot phase was used at each unit to train midwives to record data on a standardised form, to clarify terms and definitions, and to ensure data quality. These data were not included in this analysis. Consecutive women who used a birthing pool for labour were then recruited to the study between August 2002 and December 2005. Participating obstetric units were also invited to collect data for a control group comprising women who met eligibility criteria to use the birthing pool but did not do so. Eligibility for pool use varied across the units, approximately 90 percent adopting the UK guidelines (personal communication A Regalia), i.e. an uncomplicated pregnancy, 37 weeks or more gestation, singleton fetus with cephalic presentation, at low risk of complications [3]. Two units allowed women with a broader risk profile to use the pool. These included preterm labour (34-37 weeks), previous caesarean section, labour induction, meconium stained amniotic fluid, prolonged rupture of membranes or following administration of intravenous antibiotics for Group B Streptococcus (personal communication A Regalia).

All birthing pools were large enough for the woman to adopt different positions and move around. The water was deep enough to cover her abdomen when seated, and all women received one to one midwifery care.

Data were collected on women’s age, gestation, parity (nullipara or multipara), previous caesarean, labour onset (spontaneous or induced) and cervical dilatation before entering the pool, water temperature, duration of pool use, pain relief (opioid, epidural, non-pharmacological), reason for leaving the pool (if she got out prior to delivery), birth position for vaginal delivery (semi-recumbent, floating/supine, squatting, kneeling, other), caregiver hands on or off delivery technique, duration of labour, type of delivery (spontaneous vertex, operative vaginal, caesarean section), whether waterbirth or not, third stage management (physiological defined as placenta delivered by maternal effort without clamping of the cord and cutting; active defined as cord clamped and cut and oxytoxic injection with controlled cord traction to deliver the placenta; or mixed defined as placenta delivered by maternal effort, but cord clamped and cut pre-delivery), perineal outcome (intact perineum, labial or vaginal laceration only, first, second, third or fourth degree (extensive) tear, episiotomy). Perineal trauma was classified by the attending midwife and obstetrician according to international criteria agreed by the World Health Organisation (WHO) [23]. In addition, data were collected for estimated blood loss including PPH which was defined as minor up to 999 ml and major from 1000 ml, maternal and neonatal complications, and neonatal readmission within seven days. In Italy at this time length of stay following vaginal delivery was usually three days, and four days following a Caesarean section.

Data were entered onto standardised forms by the attending midwife in each obstetric unit. One midwife (GC) was responsible for data collection and collation from the individual units. Participating units sent their completed forms to GC who then checked and entered the data onto an Excel spreadsheet. GC maintained regular contact with each hospital and followed up missing or incomplete data by phone. GC liaised with the principal investigator (EB) who had overall responsibility for the Italian data as part of an international study.

Ethics approval was granted by Milan University Ethics Committee and the study was registered with the Director of Health Services at each hospital.

### Data analysis

Descriptive statistics were calculated for all women by parity for all women who used a birthing pool during labour who either had a waterbirth or left the pool and had a landbirth, and for the subgroup of women who had a waterbirth. We did not make a formal comparison between women who delivered in water with women who left the pool before delivery. This is because women who left the pool may have done so for reasons associated with the eventual outcome, such as fetal heart rate anomalies or slow progress in labour. Therefore, comparing interventions and outcomes between women who gave birth in water and women who got out of the pool to deliver on land would be potentially biased in favour of women who gave birth in water.

Appropriate measures of central tendency (mean, median) and dispersion (standard deviation, range) were calculated for continuous variables. Variables having a non-normal distribution, such as labour length and estimated blood loss, were log transformed for analysis but medians and inter-quartile ranges are presented for ease of interpretation. For the comparison between women who used a birthing pool with a control group of women who were eligible to use the pool but did not in one obstetric unit categorical data were compared using Chi square statistics and continuous data by T-tests by parity. Missing data were excluded from the analyses. Sensitivity analyses were done to test the assumption that pooling data from different units was appropriate. We removed the data for each unit in turn from the pooled sample to examine if the results for the remaining units changed substantially. Results were considered statistically significant if *p* < 0.05 in a two-tailed test. Data were analysed using SPSSX version 19.

## Results

Twenty-one units agreed to participate although some provided only limited data or stopped recruiting women early due to the pressure of following up missing data. Two of the units were excluded as there was uncertainty whether they were recruiting all eligible women. In one unit between February and October 2005, consecutive women were also recruited for a concurrent control group. These were women who met eligibility criteria to use the birthing pool but the pool was already occupied or they did not wish to use it. Data were available for analysis addressing objective (a) from 19 units involving 2,505 women who had used a birthing pool. To address objective (b) data were available for 114 women who had used a birthing pool and 459 women (controls) who did not use the pool during the same time period in one of the obstetric units. Complete data were available for most variables; missing values were less than one percent except for time in pool, length of labour, birth position, hands on or off at delivery, and perineal outcome where missing values were between 1.5 to 2.6 percent.

Characteristics of the obstetric units are shown in Additional file 1: Table S1. They reflect the average regional birth rate of Italian public obstetric units between 2002 and 2005, being a mixture of small (less than 500 births per year), medium (500 to 1,000), and larger units (over 1,000 births per year). Thirty obstetric units in Italy at this time had more than 2,500 deliveries per year. The recruitment rate varied between units due to differences in the size of units and the use of birthing pools. Sensitivity analysis found results to be broadly similar across units, with no single unit unduly influencing the pooled result.

### Descriptive results for all women who used a birthing pool during labour who either had a waterbirth or left the pool and had a landbirth

Characteristics, intrapartum events and interventions in women using the birthing pools are shown in Additional file 2: Table S2 for all women (a), and restricted to those women who had a waterbirth (b). Multiparas were older with a mean age of 33 years compared to 30 years in nulliparas. Mean gestation was term; ten women had a gestation of less than 37 weeks (minimum gestation was 34 weeks). Of the 2,505 women who used a birthing pool, 152 had their labour induced, 6.9% in nulliparas, and 4.7% in multiparas. Fifteen of the multiparas had a previous Caesarean section.

More nulliparas received interventions, such as augmentation, than multiparas. Half of the nulliparas, and three-quarters of multiparas delivered in the birthing pool. Women who left the pool before delivery generally did so at their own request although in 13% women it was due to slow progress in labour, and in 18% it was due to fetal heart rate anomalies. Multiparas were more likely to deliver in a semi-recumbent position and the midwife was more likely to adopt a ‘hands off’ the perineum and baby’s head technique at delivery than for nulliparas.

Overall, the placenta was most commonly delivered by active management, (umbilical cord clamped and cut, oxytocic injection, and placenta delivered by controlled cord traction). Physiological management (no cord clamping, no oxytocic injection and placenta delivery by maternal effort), was used comparatively rarely. Even for women who gave birth in the pool, only 12% of nulliparae and 13% of multiparae received physiological third stage management (Additional file 2: Table S2b). About a third of women overall received mixed management whereby no injection was given but the umbilical cord was clamped and cut before placental delivery. Manual removal of the placenta was necessary in only 19/2505 (0.8%) women, 12 of whom delivered in water.

Eight women (0.3%) had a third degree tear, there were no fourth degree tears and only one woman suffered both a third degree tear and an episiotomy. More multiparae than nulliparae had an intact perineum, 41% and 28%, and fewer had an episiotomy 19% and 4%, respectively.

Of the women who delivered in water, 12-3% had physiological third stage management, and more than a third of women had an intact perineum. A total of 125/2505 (5%) women had a PPH, 13 (0.5%) of these were major (≥1,000 mls) PPH the remainder being minor (500- < 1,000 mls) PPH (Additional file 2: Table S2a). For women who gave birth in water, of the 101 nulliparae who had physiological third stage none had a PPH. Of the 91 multiparae who had a physiological third stage two had a PPH, neither of which were classified as major.

Neonatal outcomes are shown in Additional file 3: Tables S3(a) for all women and (b) restricted to waterbirths. Adverse outcomes were very rare; there were no stillbirths or neonatal deaths. The two cases of umbilical cord snap occurred during waterbirth. Neither baby required resuscitation, transfer to neonatal intensive care (NICU) or required a blood transfusion, and both had Apgar scores of at least seven at one, five and ten minutes. One baby had Apgar scores of two at one minute and seven at five minutes following waterbirth, received facial oxygen and was admitted to NICU. One baby required resuscitation and had Apgar scores of six, eight and ten at one, five and ten minutes and did not require admission to NICU. Three babies with pyrexia or suspected infection were admitted to NICU following waterbirth. None required any respiratory assistance, and no infections were subsequently diagnosed. Two babies were admitted to NICU with a congenital abnormality. Of the remaining five, one was born following shoulder dystocia, two babies were admitted to NICU with respiratory difficulties (one following a birth out of water, the other following an emergency Caesarean section with a pneumonia which resolved by day three). Two babies were admitted to NICU for a few hours of observation following waterbirth, one had an Apgar less than seven at five minutes, the birth weight of the other was 2,585 grammes.

### Comparison with control group

Maternal characteristics, intrapartum events and interventions, and maternal outcomes of birthing pool users and controls are shown in Additional file 4: Table S4. The proportion of nulliparas was significantly higher for the birthing pool than the controls (61 percent compared to 44 percent) so the comparisons are shown stratified by parity. There was no evidence of a difference for maternal age, gestation, artificial rupture of membranes or augmentation.

Irrespective of parity, women who used the birthing pool were more likely to adopt an upright (semi-recumbent, squatting, kneeling, all-fours) birth position, and have hands-off delivery technique (perineum or fetal head not touched during the birth) than controls.

As almost all women had a spontaneous vertex delivery (SVD) there was no evidence of a difference in mode of delivery between nulliparae and multiparae who used the pool and the controls. A significantly higher proportion of women who used the birthing pool had a physiological third stage compared with the control group who all had active management of the third stage.

Whilst nulliparas who used the birthing pool were significantly more likely to have a spontaneous second degree perineal tear, they were significantly less likely to have an episiotomy than nulliparous controls. There was no evidence of a difference for perineal outcomes for multiparas, and no woman sustained extensive perineal trauma.

There was no evidence of a difference in the incidence of PPH which were few overall.

Adverse neonatal outcomes occurred too rarely to make comparisons.

## Discussion

The results of our prospective study of 2,505 women are reassuring: maternal outcomes were good, as would be expected in this low risk population and adverse neonatal outcomes were rare. The vast majority of women (94 percent of nulliparas and 99 percent of multiparas) had a SVD and few had extensive perineal trauma or a PPH. We also found that significantly more women who laboured in water adopted an upright birth position and had hands off delivery technique. Nulliparas who laboured in water had significantly fewer episiotomies compared with controls.

The high proportion of SVDs was similar to that seen with international studies on birthing pool use [13,14,24] and is reassuring in a country with a high overall Caesarean section rate of 38% [5].

We found very few women used pharmacological pain relief or complementary therapies such as aromatherapy or homeopathy. This is not an unusual finding for Italy where few obstetric units provide an epidural service for non-operative deliveries, and injected opioids or inhalational analgesia are not generally available.

It is thought that the progress of labour may be slowed if a woman enters the pool before her cervix has dilated to at least 4 cm [1]. We found no relation between cervical dilatation at pool entry and duration of labour in our sample of 2,505 women. The subjectivity of assessing duration of labour may contribute to the difference in results between studies. Also, cervical dilatation is just one aspect indicating labour progress-cervical effacement and application of presenting part of the fetus to the cervix are equally important.

The higher proportion of nulliparas who laboured in water and sustained a perineal tear was offset by a significant reduction in episiotomy. This is consistent with other studies [17,24,25]. Having an episiotomy precludes having a first or second spontaneous perineal tear, therefore research reporting higher rates of first and second degree tears in women not having an episiotomy is not surprising [26]. Whilst episiotomies require suturing, this is not so for all spontaneous tears. The longer term consequences of these two different types of injury are currently under-researched. The vast majority of the literature focuses on morbidity of third or fourth degree perineal trauma.

The incidence of extensive perineal tears was very low also (0.3%). Other large prospective observational studies of women who used a birthing pool in the hospital setting report higher incidences of extensive perineal trauma, ranging from 0.95% [27] to 2.3% [14,24]. A recent retrospective analysis that compared the incidence of extensive perineal trauma in women who gave birth in water with women who gave birth out of water reported a greater incidence of third degree tears for women who had a waterbirth compared with other women: 4/160 (2.5%) and 8/623 (1.2%), respectively. This difference was not significant with a wide 95% confidence interval ranging from 0.58 to 6.2 [17].

One potential explanation for the low incidence of extensive perineal trauma found in our study could be that only a small proportion of women had an operative vaginal delivery which is a known risk factor for extensive perineal trauma. In our prospective study the potential for misclassification of perineal trauma was minimised as the midwife and attending obstetrician were both involved with defining the degree of trauma at the time of delivery, and it was graded according to International criteria [23].

A factor that might influence perineal outcome is maternal birth position. In this study, significantly more pool users adopted an upright birth position. A Cochrane review evaluating effects of birth position in women without an epidural reported an increase in second degree tears, (Relative Risk (RR) 1.35, 95% CI: 1.20, 1.51) and a corresponding reduction in episiotomy (RR 0.79, 95% CI: 0.70, 0.90), in women who were in an upright position compared with women in supine positions [28]. However, the interaction between birth position and delivering in water in terms of effects on the perineum is not clear and requires further research.

A concern about waterbirth is that it may predispose women to have a PPH as typically waterbirth involves physiological management of the third stage of labour. We found a low overall incidence of PPH which was in keeping with our sample of women with an uncomplicated pregnancy. However, irrespective of land or water birth, determining whether a woman has had a PPH is based on estimation of blood loss which is inevitably an imprecise measurement. Whilst PPH is defined by estimated blood loss, it is only one of the factors taken into account in deciding whether a woman requires a blood transfusion or not. The majority of the women who gave birth in water had either active or mixed management. This was unexpected as waterbirth is usually associated with physiological management, and may reflect the influence of the predominantly medical care model in Italy.

A recent retrospective study reporting on PPH and third stage management for low risk women found an increased risk of blood loss > 1000 mls with active versus physiological third stage management (RR 2.12, 95% CI: 1.39, 3.22) [29]. This is in contrast with a Cochrane review of seven RCTs and 8,247 women that showed that active management reduced the risk of major PPH compared with physiological management [21]. However, the women in the trials were at mixed risk of excessive bleeding. Two of the RCTs were restricted to women at low risk of childbirth complications [30]. These findings were inconclusive for major PPH (RR 0.31, 95% CI: 0.05, 2.17), but there was a significant reduction in minor PPH with active management (RR 0.33, 95% CI: 0.20, 0.56). A difference in mean estimated blood loss of -78 mls (95% CI: -96, -62) was also reported but is of questionable clinical significance. It is worth noting that the components of physiological management are not always well defined and vary between studies, which may influence results. A study involving low risk women has shown that ‘holistic psychophysiological care’ in the third stage may reduce the risk of PPH [22]. This involves immediate and uninterrupted skin-to-skin contact between mother and baby following birth, encouraging her to focus on the baby, self-attachment breastfeeding, and the placenta being delivered by maternal effort and gravity without interventions. It is not clear to what extent this was practised in the units taking part in this study.

In our sample there were no serious adverse neonatal outcomes. All 10 babies admitted to NICU were discharged home with their mothers. In Italy paediatricians routinely attend childbirth, consequently there may be a lower threshold for intervention than in countries where the paediatrician is not routinely present. There were two cases of umbilical snap in this study sample. This unintended effect has been previously reported for babies born in water [14,16]. One possible reason for this could be inadvertent traction on the umbilical cord as the baby is lifted up out of the water before the placenta is delivered. Whilst a reassuringly rare event, umbilical cord snap is not unique to waterbirth and can also occur with landbirth.

The results presented here are from a large sample of women representative of state hospitals in Northern Italy. In Italy at the time of the data collection for this study (2002 to 2005) about 88 percent of women received their intrapartum care in public maternity hospitals staffed by midwives, obstetricians, paediatricians and anaesthetists [31]. Southern Italy was under-represented because, at this time, there were few obstetric units with birthing pools, and those few that did, declined to participate. This study was strengthened by prospective data collection and having a low proportion of missing data.

However, a limitation is this study is that, although all 46 obstetric units with a birthing pool were invited to participate, only 21 agreed to do so and data relating to two of them could not be used due to concerns about quality. In addition, several units stopped collecting data early due to the pressure of following up missing data, and others used their pool rarely, providing data on only small numbers of women (Additional file 1). Another potential limitation relates to the fact that the data were collected during 2002–2005. However, eligibility criteria for using a birthing pool and caregiver practice in relation to birthing pool use have not changed since this time period so the results are relevant to current practice.

Northern Italy may not be representative of Italy as a whole and is dissimilar from other parts of Europe in having a highly medicalised obstetric service. Women receive an ultrasound scan at the beginning of labour to exclude breech presentation and obstetricians usually assess all women on admission to hospital in labour before handing over care to the midwife. Obstetricians and paediatricians are also routinely present for normal birth. Rates of intervention including Caesarean section are unusually high at 38 percent [32]. Despite this, the use of birthing pools has not increased in the years since this study and most pools are under-used (personal communication A Regalia).

## Conclusions

This study shows that, for women at low risk of complications, use of a birthing pool during labour and birth is associated with lower intervention rates compared with concurrent controls. This study will reassure women who choose to use a birthing pool during labour.

## Competing interests

The authors declare that they have no competing interests.

## Authors’ contributions

JH analysed the data and drafted the manuscript. EB conceived of and designed the study, coordinated data collection, participated in interpretation of the analyses, and help draft the manuscript. AR helped with coordination of data collection. GC helped with coordination of data collection. MB participated in interpretation of the analyses, and helped draft the manuscript. LS participated in interpretation of the analyses and help draft the manuscript. All authors read and approved the final manuscript.

## Pre-publication history

The pre-publication history for this paper can be accessed here:

http://www.biomedcentral.com/1471-2393/14/17/prepub

## Supplementary Material

Additional file 1: Table S1 Study centres and recruitment time periods. Click here for file

Additional file 2: Table S2a Maternal characteristics, intrapartum events, interventions and outcomes for all women who used a birthing pool by parity; **Table S2b** maternal characteristics, intrapartum events, interventions and outcomes for women who had a waterbirth by parity. Click here for file

Additional file 3: Table S3a Neonatal outcomes for all women who used a birthing pool by parity; **Table S3b**: neonatal outcomes for women who had a waterbirth by parity. Click here for file

Additional file 4: Table S4 Maternal characteristics, intrapartum events, interventions and outcomes for women who used a birthing pool and controls who did not. Click here for file
