# Supplementary material for: Labouring women who used a birthing pool in obsteric units in Italy: prospective observational study
Source: BMC Pregnancy Childbirth. 2014 Jan 14;14:17. doi: 10.1186/1471-2393-14-17 (PMC3897991; doi:10.1186/1471-2393-14-17)
Supplement: Additional file 1: Table S1 — Study centres and recruitment time periods. [file 1471-2393-14-17-S1.doc]

Additional file Table S1. Study centres and recruitment time periods

| *N* | *Region* | *Recruitment period* | |
| --- | --- | --- | --- |
| *Small units (<500 births per year)*  *(N*=156*)* | | | |
|  | Emilia-Romagna | Jan 2004-May 2005 |  |
|  | Piedmont | Jan 2004-May 2005 |  |
|  | Veneto | May 2004-Oct 2005 |  |
| *Small-medium units (500-1,000 births per year)*  *(N*=712*)* | | |  |
|  | Lombardy | Jan 2004-May 2005 |  |
|  | Tuscany | Apr 2004-Dec 2004 |  |
|  | Marche | Feb 2004-Jan 2005 |  |
|  | Piedmont | Apr 2004-Mar 2005 |  |
|  | Emilia-Romagna | Mar-Sep 2004 |  |
| *Medium-large units (1,000-2,000 births per year)*  *(N*=1,076*)* | | |  |
|  | Tuscany | Apr 2004-Jan 2005 |  |
|  | Genova Ligury | Jul 2003-May 2005 |  |
|  | Veneto | Apr 2004-Apr 2005 |  |
|  | Lombardy | Jan 2004-Oct 2005 |  |
|  | Emilia-Romagna | Jan 2004-Oct 2005 |  |
| *Large units (>2,000 births per year)*  *(N*=561*)* | | |  |
|  | Lombardy | Aug 2002-Oct 2005 |  |
| **Total N=2,505** | | |  |
