# Supplementary material for: Labouring women who used a birthing pool in obsteric units in Italy: prospective observational study
Source: BMC Pregnancy Childbirth. 2014 Jan 14;14:17. doi: 10.1186/1471-2393-14-17 (PMC3897991; doi:10.1186/1471-2393-14-17)
Supplement: Additional file 2: Table S2a — Maternal characteristics, intrapartum events, interventions and outcomes for all women who used a birthing pool by parity; Table S2b maternal characteristics, intrapartum events, interventions and outcomes for women who had a waterbirth by parity. [file 1471-2393-14-17-S2.docx]

Additional file 2: Table S2a Maternal characteristics, intrapartum events, interventions and outcomes for all women who used a birthing pool during labour who either had a waterbirth or left the pool and had a landbirth by parity

|  | *Nulliparas*  N*=1,604 (64%)*  *[95% CI]* | *Multiparas*  N*=901(36%)*  *[95% CI]* | Overall  N=2,505 (100%)  *[95% CI]* |
| --- | --- | --- | --- |
| Age | n=1,604 | n=901 | n=2,505 |
| <20 years | 35 (2.2)  [1.6, 3.0] | 4 (0.4)  [0.2,1.1] | 39 (1.6)  [1.1, 2.1] |
| 20-29 years | 680 (42.4)  [40.0, 44.8] | 209 (23.2)  [20.6, 26.1} | 889 (35.5)  [33.6, 37.4] |
| 30-39 years | 864 (53.9)  [51.4, 56.3] | 647 (71.9)  [68.8, 74.7] | 1,511 (60.3)  [58.4, 62.2] |
| 40+ years | 23 (1.4)  [0.0, 2.1] | 40 (4.4)  [0.3, 6.0] | 63 (2.5)  [1.9, 3.2] |
| Gestation (weeks) mean (SD) | 39.5 (39.4, 39.5) | 39.5 (39.4, 39.5) | 39.5 (39.4, 39.5) |
| Labour onset | | | |
| Spontaneous | 1,494 (93.1)  [91.8, 94.3] | 859 (95.3)  [93.8, 96.5] | 2,353 (93.9)  [92.9, 94.8] |
| Induced | 110 (6.9)  [5.7, 8.2] | 42 (4.7)  [3.5, 6.2] | 152 (6.1)  [5.5, 7.5] |
| Artificial rupture of membranes | 368 (23.0)  [21.0, 25.1] | 185 (20.5)  [18.2, 23.3] | 553 (22.1)  [21.8, 25.3] |
| Augmentation | 116 (7.8)  [6.1, 8.6] | 17 (2.0)  [1.2, 3.1] | 133 (5.3)  [4.7, 6.7] |
| Mode of delivery | n=1,597 | n=900 | n=2,497 |
| SVD - land | 668 (41.8)  [39.3, 44.1] | 201 (22.3)  [19.7, 25.1] | 869 (34.8)  [32.9, 36.7] |
| SVD - water | 830 (52.0)  [49.3, 54.2] | 689 (76.6)  [73.6, 79.1] | 1,519 (60.8)  [58.9, 62.7] |
| Kristeller manoevre^§^ | 14 (0.9)  [0.5, 1.5] | 4 (0.4)  [0.2, 1.1] | 18 (0.7)  [0.4, 1.1] |
| Operative vaginal | 34 (2.1)  [1.5, 3.0] | 2 (0.2)  [0.1, 0.8] | 36 (1.4)  [1.0, 2.0] |
| Emergency Caesarean | 51 (3.2)  [2.4, 4.2] | 4 (0.4)  [0.2, 1.1] | 55 (2.2)  [1.7, 2.9] |
| Reason for leaving pool | n=768 | n=211 | n=979 |
| Maternal request | 406 (52.6)  [49.3, 56.4] | 126 (59.7)  [53.0, 66.3] | 532 (54.3)  [51.1, 57.5] |
| Slow progress 1^st^ stage | 154 (20.1)  [17.4, 23.0] | 25 (11.8)  [8.2, 16.9] | 179 (18.3)  [15.9, 20.8] |
| Slow progress 2^nd^ stage | 46 (6.4)  [4.5, 7.9] | 4 (1.9)  [0.7, 4.8] | 50 (5.1)  [3.8, 6.7] |
| Fetal concern | 121 (15.7)  [13.2, 18.5] | 37 (17.5)  [12.6, 23.3] | 158 (16.1)  [13.9, 18.6] |
| Other reason | 41 (5.3)  [3.9, 7.2] | 19 (9.0)  [5.5, 13.7] | 60 (6.1)  [4.7, 7.8] |
| Position for vaginal birth | n=1,550 | n=896 | n=2,446 |
| Upright | 964 (62.2)  [59.7, 64.6] | 677 (75.6)  [72.6, 78.3] | 1,641 (67.1)  [65.2, 68.9] |
| Lying down | 586 (37.8)  [35.4, 40.3] | 219 (24.4)  [21.7, 27.4] | 805 (32.9)  [31.0, 34.8] |
| Hands off at delivery | 800 (51.6)  [49.1, 54.1] | 617 (68.8)  [65.5, 71.5] | 1,417 (57.9)  [55.9, 59.9] |
| Third stage management | n=1,593 | n=899 | n=2,492 |
| Active | 858 (53.9)  [51.4, 56.3] | 459 (51.1)  [47.8, 54.3] | 1,317 (52.8)  [50.9, 54.8] |
| Physiological | 121 (7.6)  [6.4, 9.0] | 96 (10.7)  [8.8, 12.9] | 217 (8.7)  [7.6, 9.9] |
| ‡Mixed management | 614 (38.5)  [36.1, 40.9] | 344 (38.2)  [35.1, 41.5] | 958 (38.4)  [36.5, 40.4] |
| Perineal outcome | n=1,567 | *n*=901 | n=2,468 |
| Intact | 445 (28.4)  [25.9, 30.4] | 370 (41.1)  [37.9, 44.4] | 815 (33.0)  [31.2, 35.0] |
| Labial tear only | 64 (4.1)  [3.1, 5.1] | 29 (3.2)  [2.2, 4.6] | 93 (3.8)  [3.0, 4.6] |
| 1^st^ degree tear | 423 (26.9)  [24.6, 29.0] | 289 (32.1)  [29.1, 35.2] | 712 (28.8)  [27.1, 30.7] |
| 2^nd^ degree tear | 330 (21.0)  [18.9, 22.9] | 174 (19.3)  [16.9, 22.0] | 504 (20.4)  [18.8, 22.1] |
| 3^rd^ degree tear | 5 (0.3)  [0.1, 0.7] | 3 (0.3)  [0.1, 1.0] | 8 (0.3)  [0.1, 0.6] |
| Episiotomy | 300 (19.1)  [17.1, 20.9] | 36 (4.0)  [2.9, 5.5] | 336 (13.6)  [12.3, 15.0] |
| Postpartum haemorrhage | n=1,604 | n=901 | n=2,505 |
| Minor (500-999ml) | 80 (5.0)  [4.0, 6.2] | 32 (3.6)  [2.5, 5.0] | 112 (4.5)  [3.7, 5.3] |
| Major (≥1000 ml) | 10 (0.6)  [0.3, 1.1] | 3 (0.3)  [0.1, 1.0] | 13 (0.5)  [0.3, 0.9] |
| Manual removal of placenta | 11 (0.7)  [0.4, 1.2] | 8 (0.9)  [0.4, 1.8] | 19 (0.8) |
|  | n=1,597 | n=898 | n=2,495 |
| Time in pool mins median (inter-quartile range) | 120 (80, 150) | 75 (60, 120) | 100 (60, 140) |
|  | n=1,581 | n=896 | n=2,477 |
| Duration of labour (mins) median (inter-quartile range) | 330 (247, 430) | 194 (135, 270) | 280 (190, 386) |

Notes: CI = confidence interval; n=number analysed; *§* Kristeller manoeuvre = fundal pressure exerted in the second stage of labour to expedite delivery; augmentation by means of intravenous infusion of oxytocin; upright = semi-recumbent, squatting, standing, on knees, all fours, on birth stool; lying down = right or left lateral, supine, lithotomy; ‡mixed management = placenta delivered by maternal effort, but cord clamped and cut pre-delivery

Additional file 2: Table 2b Maternal characteristics, intrapartum events, interventions and outcomes for **the subgroup of women who had a waterbirth** by parity

|  | *Nulliparas*  N*=830 (55%)*  *[95% CI]* | *Multiparas*  N*=689 (45%)*  *[95% CI]* | *Overall*  *N=1,519 (100%)*  *[95% CI]* |
| --- | --- | --- | --- |
| Age | n=829 | n=688 | n=1,517 |
| <20 years | 14 (1.7)  [1.0, 2.8] | 4 (0.6)  [0.2,1.5] | 18 (1.2)  [0.7, 1.9] |
| 20-29 years | 348 (42.0)  [38.6, 45.3] | 148 (21.5)  [18.6, 24.7} | 496 (32.7)  [30.3, 35.1] |
| 30-39 years | 456 (55.0)  [51.4, 58.3] | 502 (73.0)  [69.4, 76.1] | 967 (63.7)  [61.3, 66.2] |
| 40+ years | 11 (1.3)  [0.7, 2.4] | 34 (4.9)  [3.6, 6.8] | 45 (3.0)  [2.2, 3.9] |
|  | *n*=830 | *n*=689 | n=1,519 |
| Gestation (weeks) mean (SD) | 39.4 (39.3, 39.5) | 39.5 (39.4, 39.6) |  |
| Labour onset | | | |
| Spontaneous | 790 (95.2)  [93.5, 96.4] | 657 (95.4)  [93.5, 96.7] | 1,447 (95.3)  [94.1, 96.3] |
| Induced | 40 (4.8)  [3.6, 6.5] | 32 (4.6)  [3.3, 6.5] | 72 (4.7)  [3.7, 5.9] |
| Artificial rupture of membranes | 155 (18.7)  [16.2, 21.5] | 129 (18.7)  [16.0, 21.8] | 284 (18.7)  [16.8, 20.7] |
| Position for waterbirth | | | |
| Upright | 730 (87.9)  [85.5, 90.1] | 593 (86.1)  [83.3, 88.6] | 1,323 (87.1)  [85.3, 88.7] |
| Lying down | 100 (12.0)  [9.9, 14.6] | 96 (13.9)  [11.4, 16.7] | 196 (12.9)  [12.9, 16.8] |
| Hands off at delivery | 653 (78.7)  [75.8, 81.3] | 550 (79.8)  [76.7, 82.7] | 1,203 (79.2)  [89.2, 92.4] |
| Third stage management | *n*=827 | *n*=688 | n=1,515 |
| Active | 309 (37.4)  [34.1, 40.7] | 305 (44.3)  [40.7, 48.1] | 614 (40.5)  [38.0, 43.0] |
| Physiological | 101 (12.2)  [10.2, 14.6] | 91 (13.0)  [10.9, 16.0] | 192 (12.7)  [11.0, 14.4] |
| ‡Mixed management | 417 (50.2)  [46.8, 53.7] | 292 (42.3)  [38.7, 46.2] | 709 (46.8)  [44.3, 49.3] |
| Perineal outcome | *n*=830 | *n*=689 | n=1,519 |
| Intact | 294 (35.4)  [32.2, 38.7] | 294 (42.7)  [39.1, 46.5] | 588 (38.7)  [36.2,41.2] |
| Labial tear only | 43 (5.2)  [3.8, 6.9] | 21 (3.0)  [1.9, 4.6] | 64 (4.2)  [3.2, 5.3] |
| 1^st^ degree tear | 284 (34.2)  [31.1, 37.5] | 231 (33.5)  [30.2, 37.2] | 515 (33.9)  [31.5, 36.3] |
| 2^nd^ degree tear | 194 (23.4)  [20.6, 26.4] | 140 (20.3)  [17.5, 23.5] | 334 (22.0)  [19.9, 24.1] |
| 3^rd^ degree tear | 3 (0.4)  [0.1, 1.1] | 1 (0.1)  [0.0, 0.1] | 4 (0.3)  [0.1, 0.8] |
| Episiotomy | 12 (1.4)  [0.8, 2.5] | 4 (0.6)  [0.2, 1.5] | 16 (1.1)  [0.6, 1.7] |
| Postpartum haemorrhage | | | |
| Minor (500-999 ml) | 22 (2.7)  [1.8, 4.0] | 21 (3.0)  [2.0, 4.6] | 43 (2.8)  [2.0, 3.8] |
| Major (≥1000 ml) | 5 (0.6)  [0.2, 1.4] | 2 (0.3)  [0.1, 1.0] | 7 (0.5)  [0.2, 1.0] |
| Manual removal of placenta | 5 (0.6)  [0.2, 1.4] | 7 (1.0)  [0.5, 2.1] | 12 (0.8)  [0.4, 1.4] |
|  | n=825 | n=686 | n=1,511 |
| Time in pool mins  median (inter-quartile range) | 120 (80, 160) | 70 (60, 110) | 90 (60, 140) |
|  | n=825 | n=687 | n=1,517 |
| Duration of labour (mins) median (inter-quartile range) | 297 (225, 385) | 180 (121, 241) | 240 (165, 330) |

Notes: CI = confidence interval; n=number analysed; *§* Kristeller manoeuvre = fundal pressure exerted in the second stage of labour to expedite delivery; augmentation by means of intravenous infusion of oxytocin; upright = semi-recumbent, squatting, standing, on knees, all fours, on birth stool; lying down = right or left lateral, supine, lithotomy; ‡mixed management = placenta delivered by maternal effort, but cord clamped and cut pre-delivery
