# Supplementary material for: Labouring women who used a birthing pool in obsteric units in Italy: prospective observational study
Source: BMC Pregnancy Childbirth. 2014 Jan 14;14:17. doi: 10.1186/1471-2393-14-17 (PMC3897991; doi:10.1186/1471-2393-14-17)
Supplement: Additional file 3: Table S3a — Neonatal outcomes for all women who used a birthing pool by parity; Table S3b: neonatal outcomes for women who had a waterbirth by parity. [file 1471-2393-14-17-S3.docx]

Additional file 3 Table S3a neonatal outcomes for all women who used a birthing pool during labour who either had a waterbirth or left the pool and had a landbirth

|  | *Nulliparas*  N*=1,604 (64%)*  *[95% CI]* | *Multiparas*  N*=901(36%)*  *[95% CI]* | *Overall*  *N=2,505 (100%)*  *[95% CI]* |
| --- | --- | --- | --- |
|  | n=1,604 | n=901 | *N=2,505* |
| Umbilical cord snap | 1 (0.1)  [0.0, 0.4] | 1 (0.1)  [0.0, 0.4] | 2 (0.1)  [0.0, 0.3] |
|  | n=1,591 | n=896 | 2,487 |
| Apgar <7 at 1 minute | 34 (2.1)  [1.5, 3.0] | 13 (1.5)  [0.8, 2.5] | 47 (1.9)  [1.4, 2.5] |
|  | n=1,591 | n=897 | n=2,488 |
| Apgar <7 at 5 minutes | 1 (0.1)  [0.0, 0.4] | 0 | 1 (0.04)  [0.0, 0.2] |
| Facial oxygen only | 10 (0.6)  [0.3, 1.1] | 0 | 10 (0.4)  [0.2, 0.7] |
| Admission to NICU | 6 (0.4)  [0.2, 0.8] | 4 (0.4)  [0.2, 1.1] | 10 (0.4)  [0.2, 0.7] |
|  | n=1,592 | n=897 | n=2,489 |
| Birth weight (g) mean [95% CI] | 3,326  [3,307, 3,344] | 3,436  [3,410, 3,462] | 3,365  [3,350, 3,381] |

Notes: CI = confidence interval; n=number analysed

Table 3b neonatal outcomes for the **subgroup of** **women who had a waterbirth**

|  | *Nulliparas*  N*=830 (54.6)*  *[95% CI]* | *Multiparas*  N*=689 (45.3)*  *[95% CI]* | *Overall*  *N=1,519 (100%)*  *[95% CI]* |
| --- | --- | --- | --- |
|  | n=829 | n=688 | n=1,517 |
| Umbilical cord snap | 1 (0.1)  [0.0, 0.4] | 1 (0.1)  [0.0, 0.4] | 2 (0.1)  [0.0, 0.5] |
| Apgar <7 at 1 minute | 9 (1.1)  [0.6, 2.0] | 8 (1.2)  [0.6, 2.3] | 17 (1.1)  [0.7, 1.8] |
|  | n=821 | n=684 | n=1,505 |
| Apgar <7 at 5 minutes | 1 (0.1)  [0.0, 0.7] | 0 | 1 (0.1)  [0.0, 0.4] |
| Facial oxygen only | 3 (0.4)  [0.1, 1.0] | 1 (0.1)  [0.0, 0.8] | 4 (0.3)  [0.0, 0.8] |
| Admission to NICU | 5 (0.6)  [0.3, 1.5] | 3 (0.4)  [0.2, 1.3] | 8 (0.5)  [0.2, 1.0] |
|  | n=822 | n=685 | n=1,507 |
| Birth weight (g) mean [95% CI] | 3,294  [3,269, 3,319] | 3,426  [3,296, 3,456] | 3,354  [3,335, 3,373] |
