# Supplementary material for: Labouring women who used a birthing pool in obsteric units in Italy: prospective observational study
Source: BMC Pregnancy Childbirth. 2014 Jan 14;14:17. doi: 10.1186/1471-2393-14-17 (PMC3897991; doi:10.1186/1471-2393-14-17)
Supplement: Additional file 4: Table S4 — Maternal characteristics, intrapartum events, interventions and outcomes for women who used a birthing pool and controls who did not. [file 1471-2393-14-17-S4.docx]

Additional file 4 Table S4: maternal characteristics, intrapartum events, interventions and outcomes for all women who used a birthing pool during labour who either had a waterbirth or left the pool and had a landbirth and controls who did not use a birthing pool

|  | *Nullips*  *Birthing pool users (*N*=70)* | *Nullips*  *Controls*  *(*N*=202)* | *p-value* | *Multips*  *Birthing pool users (*N*=44)* | *Multips*  *Controls*  *(*N*=257)* | *p-value* |
| --- | --- | --- | --- | --- | --- | --- |
| Age (years) mean [95% CI] | 30.1  [29.0, 31.3] | 30.1  [29.4, 30.7] | 0.962 | 33.0  [29.7, 34.2] | 33.6  [29.1, 34.2] | 0.333 |
| Gestation (weeks) mean [95% CI] | 39.5  [0.2, 39.7] | 39.6  [0.4, 39.7] | 0.574 | 39.6  [0.3, 40.0] | 39.4  [0.3, 39.6] | 0.273 |
| Artificial rupture of membranes | 17 (24.3)  [15.8, 35.5] | 32 (15.8)  [11.5, 21.5] | 0.113 | 3 (6.8)  [2.4, 18.2] | 43 (16.0)  [12.7, 21.8] | 0.091 |
| Augmentation | 10 (14.3)  [8.0, 24.3] | 32 (15.8)  [11.5, 21.5] | 0.756 | 1 (2.3)  [0.4, 11.8] | 18 (7.0)  [4.5, 10.8] | 0.233 |
| Position for birth | n=68 | n=195 |  | n=44 | n=252 |  |
| Upright | 27 (39.7)  [28.0, 52.3] | 13 (6.6)  [4.3, 13.1] | 0.000 | 31 (70.5)  [54.8, 83.2] | 29 (11.5)  [7.8, 16.1] | 0.000 |
| Lying down | 38 (55.9)  [42.7, 65.4] | 180 (92.3)  [87.7, 95.3] |  | 10 (22.7)  [12.8, 37.0] | 214 (84.9)  [78.2, 87.3] |  |
| Other | 3 (4.4)  [1.5, 11.9] | 2 (1.0)  [0.3, 3.6] |  | 3 (6.8)  [2.4, 18.2] | 9 (3.6)  [1.9, 6.5] |  |
|  | n=69 | n=202 |  | n=44 | n=257 |  |
| Hands off at delivery | 28 (40.6)  [29.8, 52.4] | 18 (8.9)  [5.4, 13.7] | 0.000 | 30 (68.2)  [53.4, 80.0] | 30 (11.7)  [8.3, 16.2) | 0.000 |
| Mode of delivery | n=70 | n=201 |  | n=44 | n=257 |  |
| SVD | 68 (97.1)  [90.0, 99.2] | 191 (95.0)  [91.1, 97.3] | 0.736 | 44 (100)  [92.0, 100] | 256 (99.6)  [87.8, 94.6] | 0.679 |
| ^§^Kristellor | 0 | 2 (1.0)  [0.3, 3.6] |  | 0 | 0 |  |
| Operative vaginal | 1 (1.4)  [0.3, 7.8] | 2 (1.0)  [0.3, 3.6] |  | 0 | 0 |  |
| Emergency caesarean section | 1 (1.4)  [0.3, 7.8] | 6 (3.0)  [1.4, 6.4] |  | 0 | 1 (0.4)  [0.0, 2.2] |  |
| Third stage management | n=69 | n=202 |  | n=44 | n=257 |  |
| Active | 49 (71.0)  [59.4, 80.4] | 202 (100)  [98.1, 100] | 0.736 | 21 (47.7)  [33.8, 62.1] | 257 (100)  [98.5, 100] | 0.679 |
| Physiological | 14 (20.3)  [12.5, 31.2] | 0 |  | 16 (36.4)  [23.8, 51.1] | 0 |  |
| Mixed | 6 (8.7)  [4.1, 17.7] | 0 |  | 7 (15.9)  [7.9, 29.4] | 0 |  |
| Perineal outcome | n=69 | n=202 |  | n=44 | n=257 |  |
| Intact | 11 (15.9)  [9.0, 26.8] | 40 (19.8)  [14.9, 25.8] | 0.009 | 14 (31.8)  [20.0,46.6] | 102 (39.7)  [33.9, 45.8] | 0.140 |
| Labial tear only | 3 (4.3)  [0.9, 26.8] | 1 (0.5)  [0.0, 2.7] |  | 1 (2.3)  [2.2, 12.0] | 3 (1.2)  [0.2, 3.4] |  |
| 1^st^ degree tear | 14 (20.3)  [12.5, 31.2] | 24 (11.9)  [8.1, 17.1] |  | 14 (31.8)  [20.0, 46.6] | 53 (20.6)  [16.1, 26.0] |  |
| 2^nd^ degree tear* | 18 (26.1)  [16.9, 37.0] | 30 (14.9)  [10.6, 20.4] |  | 12 (27.3)  [16.3, 41.9] | 52 (20.2)  [15.8, 25.6] |  |
| Episiotomy | 23 (33.3)  [23.0, 44.5] | 107 (53.0)  [46.1, 59.7] |  | 3 (6.8)  [2.4, 18.2] | 47 (18.3)  [14.0, 23.5] |  |
| PPH | n=70 | n=202 |  | n=44 | n=257 |  |
| minor (500-999 ml) | 3 (4.3)  [1.5, 11.9] | 21 (10.4)  [7.0, 15.4] | 0.204 | 2 (4.5)  [1.3, 15.1] | 12 (4.7)  [2.7, 8.0] | 0.841 |
| major (≥1000 ml) | 0 | 2 (1.0)  [0.3, 3.7] |  | 1 (2.3)  [1.3, 15.1] | 3 (1.2)  [0.6, 3.9] |  |
|  | n=70 | n=202 |  | n=44 | n=257 |  |
| Manual removal of placenta | 1 (1.4)  [0.3, 7.8] | 1 (0.5)  [0.1, 2.8] | 0.431 | 1 (2.3)  [0.4, 11.8] | 4 (1.6)  [0.6, 3.9] | 0.731 |
| Duration of labor (mins) median  (inter-quartile range) | 326  [218, 449.5] | 348  [248, 445] | 0.448 | 180  [115, 296] | 156  [110, 215] | 0.056 |
|  | n=70 | n=202 |  | n=44 | n=257 |  |
| Birth weight (grammes) mean  [95% CI] | 3,304  [,3216, 3,392] | 3,286  [,3237, 3,335] | 0.717 | 3,400  [3,286, 3,514] | 3,352  [3,307, 3,397] | 0.427 |

*Notes:* Notes: CI = confidence interval; n=number analysed; ^§^Kristellor = fundal pressure exerted in the second stage of labour to expedite delivery. Upright= semi-recumbent, squatting, standing, on knees, all fours, on birth stool; Lying down= right or left lateral, supine, lithotomy.
